# Characterization of radiotherapy component impact on MR imaging quality for an MRgRT system

**DOI:** 10.1002/acm2.13054

**Published:** 2020-11-19

**Authors:** Benjamin C. Lewis, Bruce Gu, Ryan Klett, Rajiv Lotey, Olga L. Green, Taeho Kim

**Affiliations:** ^1^ Department of Radiation Oncology Washington University School of Medicine St Louis MO USA; ^2^ BJC HealthCare St Louis MO USA; ^3^ ViewRay Inc Oakwood Village OH USA

**Keywords:** MRgRT, MR‐LINAC, MRI isocenter variation

## Abstract

Radiotherapy components of an magnetic resonnace‐guided radiotherapy (MRgRT) system can alter the magnetic fields, causing spatial distortion and image deformation, altering imaging and radiation isocenter coincidence and the accuracy of dose calculations. This work presents a characterization of radiotherapy component impact on MR imaging quality in terms of imaging isocenter variation and spatial integrity changes on a 0.35T MRgRT system, pre‐ and postupgrade of the system. The impact of gantry position, MLC field size, and treatment table power state on imaging isocenter and spatial integrity were investigated. A spatial integrity phantom was used for all tests. Images were acquired for gantry angles 0–330° at 30° increments to assess the impact of gantry position. For MLC and table power state tests all images were acquired at the home gantry position (330°). MLC field sizes ranged from 1.66 to 27.4 cm edge length square fields. Imaging isocenter shift caused by gantry position was reduced from 1.7 mm at gantry 150° preupgrade to 0.9 mm at gantry 120° postupgrade. Maximum spatial integrity errors were 0.5 mm or less pre‐ and postupgrade for all gantry angles, MLC field sizes, and treatment table power states. However, when the treatment table was powered on, there was significant reduction in SNR. This study showed that gantry position can impact imaging isocenter, but spatial integrity errors were not dependent on gantry position, MLC field size, or treatment table power state. Significant isocenter variation, while reduced postupgrade, is cause for further investigation.

## 1 INTRODUCTION

Magnetic resonance‐guided radiotherapy (MRgRT) with combined MR‐RT systems provide many benefits, including superior soft tissue contrast without using ionizing radiation for patient positioning, real‐time tumor tracking, beam gating, and monitoring of diseased and normal tissue over the course of radiotherapy.^1^, ^2^, ^3^, ^4^, ^5^, ^6^ Online treatment plan adaptation with excellent local tumor control and limited toxicity has been demonstrated using MRgRT.^7^, ^8^, ^9^, ^10^ Benefits provided by MRgRT systems are dependent on consistent and accurate MRI. However, magnetic fields, including the B0 field, gradient fields, and RF pulses, can be impacted by eddy currents, imperfect shimming, and nonlinear gradients.^11^, ^12^, ^13^ Image distortion or artifacts could lead to imperfect adaptive treatment planning, inaccurate dose calculation and poor patient alignment.^12^, ^13^, ^14^

Many components of the LINAC and beam delivery systems have the potential to further reduce the accuracy of the integrated MRI system. The impact of gantry position on radiation‐MR isocenter coincidence has been investigated on MR‐^60^Co and MR‐LINAC systems, and shown variations in the MR isocenter depending on gantry position.^15^, ^16^ Utilizing an MLC for beam shaping introduces mobile metallic material, which is often the RT component closest to the magnet, that may also impact the magnetic field. A computational study by Kolling et al investigated MLC impact on field homogeneity including source‐to‐isocenter distance, field size, magnetic field strength, and other properties and found that MLC field size caused dynamic changes in field distribution.^17^ A third component impacting MRI system performance is the treatment couch, specifically the motors used to move the patient in and out of the bore and accurately align them to treatment position. These motors introduce electric current at the head and foot of the MRI bore. In MRgRT system design, attempts to reduce the impact of the gantry and LINAC systems on the MRI have been made. These changes include integrating the cryostat into the Faraday cage, utilizing carbon fiber components for RF absorption, placing electronic components outside of the radiation window, utilizing magnetically shielded rings between the MRI and LINAC, and physically moving the LINAC components away from the MRI.^18^, ^19^ Unfortunately, interactions between the MRI system and LINAC system are still present.

The authors present a phantom‐based assessment of radiotherapy component impact on the MRI system by tracking changes in imaging spatial integrity and imaging isocenter. Images were acquired pre‐ and postupgrade to the 2.0 system of an institutional MR‐LINAC system with RT system components in various positions.

## 2 MATERIALS AND METHODS

Magnetic resonance imaging isocenter variation was quantified using a phantom‐based method. Images were acquired in MRI QA mode for image center‐based quantification and assessed spatial integrity changes. Gantry position, MLC field size and treatment table power state were investigated with this image center‐based method. All experiments were conducted on the 0.35T ViewRay MRIdian MR‐LINAC system (Mountain View, CA) pre‐ and postupgrade to the 2.0 hardware system and 5.3.0 software system.

### 2.A MRIdian system

**Fig. 1:**
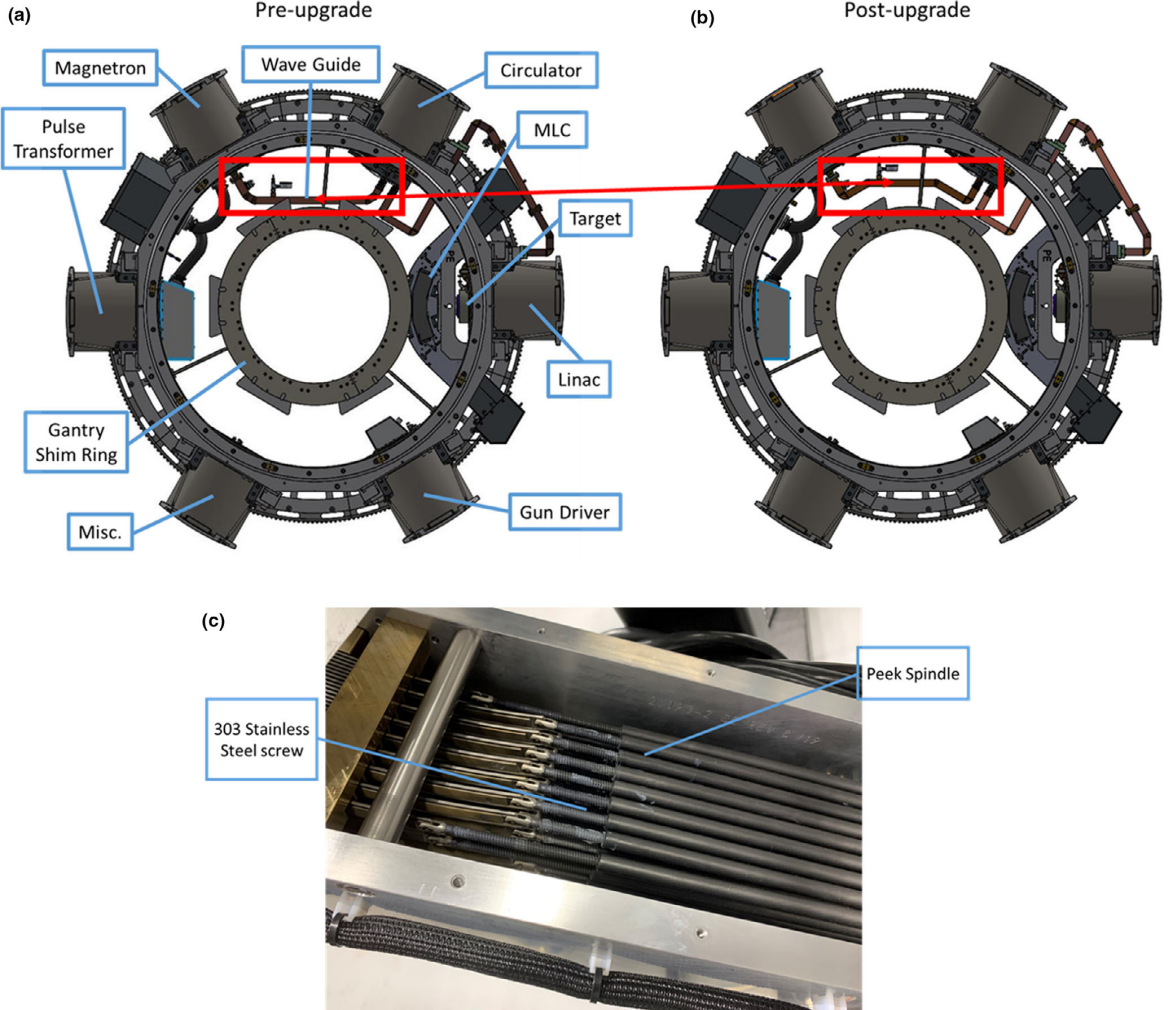
MRIdian schematic. MRIdian MR‐LINAC gantry ring preupgrade (a), and postupgrade (b). The red box and red arrow indicate the altered wave guide from the magnetron to the LINAC. Blue boxes indicate major components of the MR‐LINAC system that did not change position. (c) shows the drive screws made of 303 stainless steel that were installed in the MLC during the upgrade.

The MRIdian system utilizes a single energy 6‐MV‐flattening filter free LINAC on a gantry, with six drums containing electrical and RF components, positioned between the two halves of a 0.35T split bore Siemens MRI scanner. The LINAC system uses a double‐stacked double‐focused 138‐leaf MLC for radiation field definition. The upgrade consisted of major changes to the LINAC hardware, including the waveguide and MLC system, and system software, but did not make hardware changes to the magnet, however, significant software updates were implemented to improve shimming algorithms. The waveguide position was altered to run further from the interior of the magnet, and the MLC leaf speed was increased from 1.5 to 4.0 cm/s. The altered waveguide position is intended to reduce eddy currents produced during imaging. MLC upgrades included an improved drive mechanism with 303 stainless steel drive screws to increase leaf speed. CAD images of the gantry ring pre‐ and postupgrade, as well as the upgraded MLC components are shown in Fig. 1.

### 2.B Image center‐based quantification

**Fig. 2:**
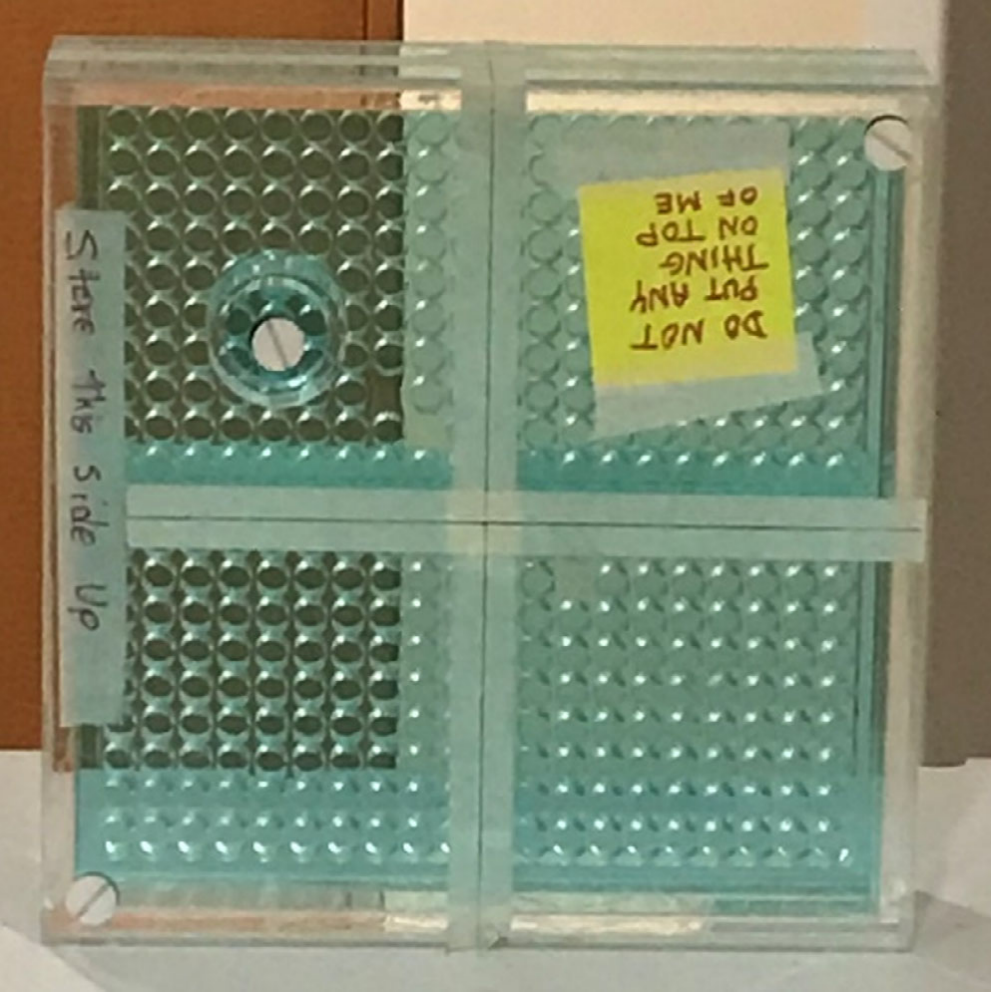
Spatial Integrity Phantom. Fluke 76‐907 uniformity and linearity water phantom used for spatial integrity and image center‐based MRI isocenter variation.

Three‐dimensional volumetric images of a Fluke 76‐907 uniformity and linearity water phantom (SI phantom) doped with 15‐mM CuSO_4_ (HP Manufacturing, Cleveland, OH) were acquired using the ViewRay recommended sequence for their spatial integrity software in MRI QA mode, shown in Fig. 2. This sequence is a clinical protocol TrueFISP sequence with TR/TE: 3.4/1.4 ms, flip angle: 60°, rBW: 534 Hz/pixel, FOV: 349 × 349 × 120 mm^3^, imaging matrix 234 × 234 × 80, voxel size: 1.5 × 1.5 × 3 mm^3^. The preupgrade images utilized the MRI gradient offset (MRI‐GO) shim mode, which used predetermined phantom‐based shim adjustments depending on gantry angle.^20^ Postupgrade images were acquired using two different shimming modes. First, the “tune‐up” shim mode, which used a single predetermined shim adjustment acquired at the home gantry position, applied to all gantry positions. In this upgrade, the predetermined shim adjustment has no additional shimming. The second was the “standard” shim, based on a 3D phase map to correct magnetic field inhomogeneity and acquired prior to image acquisition at each gantry angle. The phantom was setup in three orientations (axial, coronal, and sagittal) to provide isocenter shift quantification in the superior–inferior, anterior–posterior, and left–right directions, without torso coils in place. Images were analyzed using ViewRay’s SpatialIntegrityAnalysis 2D software. The proprietary software is purpose built for the specified phantom and pulse sequence and performs automatic rotation and translation corrections to account for setup errors. The weighted centroid of each circular marker in the grid was compared to the expected location, designated by a binary template within two analysis spheres of 100‐mm and 175‐mm radii and reported as a mean and standard deviation of positional error as a metric for spatial integrity. Output values of the software have a submillimeter accuracy using image interpolation. This software is used routinely in our clinic for monthly and annual QA to track changes in imaging quality. Isocenter shift was calculated using position of the four central markers and comparing their coordinates to the reference gantry position (0° preupgrade and 300° postupgrade).

### 2.C Radiotherapy system components

#### 2.C.1 Gantry

Impact of gantry position on imaging isocenter and imaging spatial integrity were tested using the image center‐based method. Images were acquired for each method at gantry angles 0°–330° in intervals of 30°. These tests were performed both pre‐ and postupgrade.

#### 2.C.2 MLC

MLC position impact on the MR system was investigated using only the image center‐based method on the postupgrade system. Images used for setup and adaptive planning are acquired with the jaws retracted, but cine acquisitions for target tracking and gating can be acquired when the MLCs are in treatment position. Prior to image acquisition the MRI system and treatment delivery system (TDS) were disconnected from one another to allow constant image acquisition by the MR system while the TDS was independent. Three‐dimensional volumetric TrueFISP images were acquired at the reference gantry angle for square fields with edge lengths of 1.66, 4.1, 4.16, 5.82, 9.96, 18.26, and 22.42 cm, and rectangular field of 27.4 × 24.07 cm^2^, corresponding to the largest MLC defined field available.

#### 2.C.3 Treatment table

For assessing the impact of treatment table power state on imaging isocenter and spatial integrity the image center‐based method was used on the postupgrade system. The treatment table is automatically turned off for all image acquisitions in RT mode, requiring the MRI system and TDS to be disconnected for this test so that table power shut off could be prevented.

## 3 RESULTS

### 3.A Gantry impact on 3D MRI

**Fig. 3:**
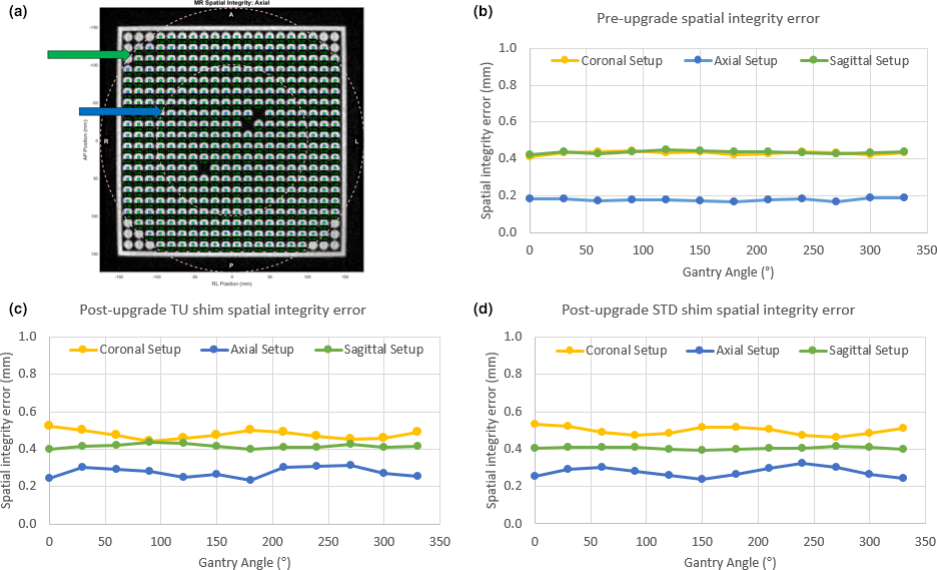
Spatial integrity variation. Image center‐based variation over gantry angle. (a) SpatialIntegryAnalysis2D software output of the SI phantom, with the blue and green arrows indicating the 100 and 175 mm analysis regions, respectively. The spatial integrity analysis mean error for each gantry angle preupgrade (b), postupgrade with the tune‐up (TU) shim (c), and postupgrade with the standard (STD) shim (d).

**Fig. 4:**
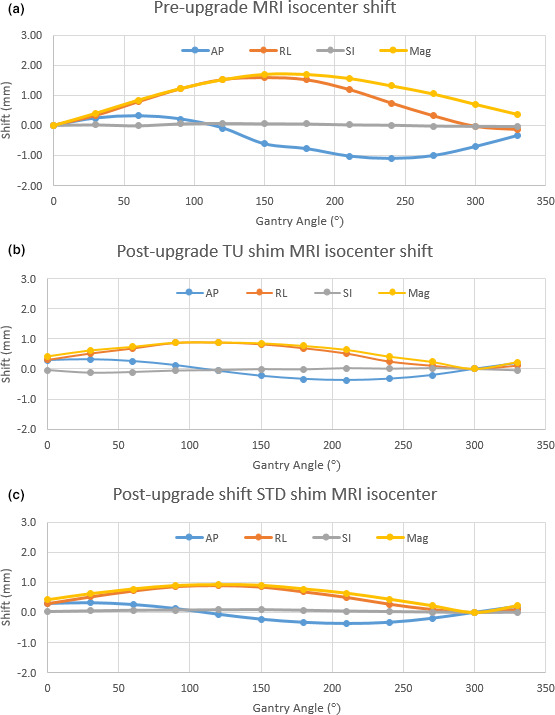
Isocenter variation. Image center‐based quantification of MRI isocenter variation from the SpatialIntegrityAnalysis2D software for preupgrade (a), postupgrade tune‐up shimming (b), and postupgrade standard shimming (c).

Volumetric 3D images of the SI phantom were acquired in the axial, coronal, and sagittal orientations [Fig. 3(a)]. Preupgrade, spatial integrity errors averaged across gantry angles were 0.18 ± 0.01 mm, 0.43 ± 0.01 mm, and 0.44 ± 0.01 mm (mean ± standard deviation) for the axial, coronal, and sagittal planes. Postupgrade utilizing the tune‐up shim method, spatial integrity errors were 0.28 ± 0.03 mm, 0.48 ± 0.02 mm, and 0.42 ± 0.01 mm for the axial, coronal, and sagittal planes. Postupgrade utilizing the standard shim method, spatial integrity errors were 0.28 ± 0.03 mm, 0.50 ± 0.02 mm, and 0.41 ± 0.01 mm. Spatial integrity errors are plotted with respect to gantry angle in Figs. 3(b)–3(d). All points passed the test criteria of the SpatialIntegrityAnalysis2D software, which uses a threshold of 1 mm error within a 100‐mm radius of isocenter and a 2‐mm error threshold within a 175‐mm radius. The isocenter identified by the SpatialIntegrityAnalysis2D software was plotted with respect to the gantry angle for preupgrade, and postupgrade with the tune‐up and standard shim modes in Fig. 4. Preupgrade, the greatest magnitude isocenter shift occurred at gantry 150°, deviating 1.7 mm. Postupgrade the largest magnitude deviations was 0.9 mm at gantry 120° for both the tune‐up and standard shim modes. The greatest planar deviation occurred in the lateral plane.

### 3.B MLC impact on 3D MRI

Volumetric 3D images were acquired of the SI phantom with the MRI system disconnected from the TDS. The mean error for all MLC positions was 0.42 ± 0.004 mm (mean ± standard deviation), compared to the mean error with the MLC completely open of 0.42 ± 0.002 mm. The maximum isocenter shift was 0.081 mm for all MLC field sizes when compared to the default open field. Visual inspection of images showed no perceptible change in image quality. Spatial integrity errors are shown in Table 1.

**Table 1:** Mean spatial integrity error and magnitude isocenter shift with the treatment table powered off and on, and at multiple MLC square field edge length.

	Table off	Table on	MLC square field edge length (cm)							
			1.66	4.1	4.16	5.82	9.96	18.26	22.42	27.4
SI error (mm)	0.42	0.48	0.42	0.42	0.42	0.42	0.41	0.42	0.42	0.42
Isocenter shift (mm)	–	0.03	0.06	0.05	0.06	0.06	0.06	0.06	0.06	0.06

### 3.C Treatment table impact on 3D MRI

**Fig. 5:**
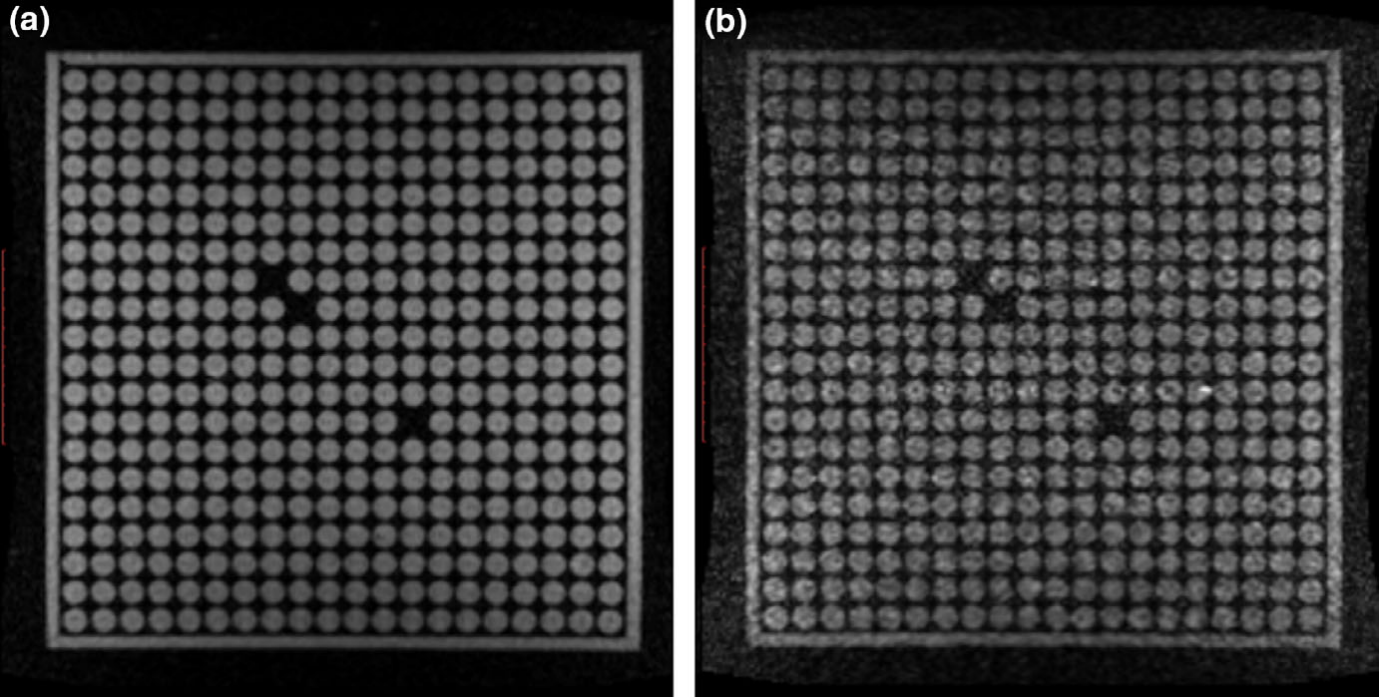
Image quality with table power. Spatial integrity phantom images acquired with the table off (a), and with the table on (b).

When the table was powered on the mean spatial integrity error increased to 0.48 ± 0.017 mm from 0.42 ± 0.02 mm with the power on. The isocenter shift was 0.03 mm with the table powered on compared to when powered off. In addition to increased spatial integrity mean error, the SNR was significantly reduced when treatment table power was enabled, from 19.41 without power to 9.04 with power. Images acquired with and without the treatment table enabled are shown in Fig. 5.

## 4 DISCUSSION

The change in MRI isocenter at multiple gantry angles, MLC field sizes, and different treatment table power states after an extensive system upgrade to an institutional 0.35T MR‐LINAC system was characterized using an image center‐based method. The MRI changes with gantry angle investigated in this study were similar to those reported by Kim et al., however, this work intended to set forth a methodology that was easily reproduced by other institutions and did not require additional tools. Their methods utilized additional phantoms, radiochromic film, and in‐house software for analysis. The image center‐based method used in this work utilized pulse sequences, a phantom and software readily available to users of the ViewRay MRIdian system, and routinely used in machine QA or patient treatment. This provides the ability for other centers to assess and characterize institutional systems without acquiring additional phantoms or developing in‐house analysis tools.

All MRI isocenter shift values presented in this study are relative to the system home gantry position, designated at installation or upgrade. Preupgrade this was at gantry 0°, and postupgrade at gantry 330°. With the system upgrade removing the MRI‐GO shimming system there was concern that the new single shim method would be insufficient to compensate for the rotation of the gantry around the MR magnet. However, the analysis presented in this work shows that the magnitude MRI isocenter shifts were reduced from 1.7 to 0.9 mm measured with the image center‐based method.

Conversely to the improvement in MRI isocenter variation, spatial integrity degraded slightly after the upgrade, increasing from 0.18 to 0.28 mm and 0.43 to 0.48 mm in the axial and coronal planes, respectively, but decreasing from 0.44 to 0.41 mm in the sagittal plane. The mean spatial integrity errors remained consistent across gantry angles.

This work had some limitations, the first being the limited image resolution due to gradient performance, SNR, and clinical protocols. The image resolution was altered by the SpatialIntegrityAnalysis2D software, which interpolated the data prior to analysis. Prior studies have utilized MRI image acquisitions with pixel edge lengths of 1.5 mm or greater to report on submillimeter average geometric distortion values for quality assurance and pulse sequence design, including studies using an identical phantom to that used in this study.^16^, ^19^, ^20^, ^21^, ^22^ In addition, the phantom used in this study cannot replicate the magnetic susceptibility or imaging volume of patient anatomy. The larger size of a patient and increased magnetic susceptibility could further increase the inhomogeneities of the main magnetic field, gradient fields, and RF pulses.

This work demonstrates that the MRI isocenter remains dependent on gantry angle after a significant vendor upgrade due to the significant effects of the ferromagnetic gantry on the MRI system altering field homogeneity. Isocenter deviation remained consistent in magnitude between the standard and tune‐up shimming methods postupgrade indicating that reacquisition of the 3D phase map at each gantry angle was not sufficient to correct for magnetic field inhomogeneities. MLC field size and treatment table power did not significantly impact the imaging isocenter or spatial integrity of the MR system.

## 5 CONCLUSION

MRI isocenter variation and spatial integrity variations were characterized pre‐ and postupgrade of an institutional 0.35T MRIdian MR‐LINAC system to the 2.0 version, and 5.3.0 software version. The method presented resulted in isocenter shifts with maximum magnitude less than 1 mm, with tools readily available, and methods reproducible, by institutions with the MRIdian ^60^Co and LINAC systems. The MRI isocenter variation postupgrade is under 1 mm but warrants continued investigation for sources and improvement.
